# Expulsion of Trichuris muris is associated with increased expression of angiogenin 4 in the gut and increased acidity of mucins within the goblet cell

**DOI:** 10.1186/1471-2164-10-492

**Published:** 2009-10-24

**Authors:** Riccardo D'Elia, Matthew L deSchoolmeester, Leo AH Zeef, Steven H Wright, Alan D Pemberton, Kathryn J Else

**Affiliations:** 1 Faculty of Life Sciences, University of Manchester, Manchester, M13 9PT, UK; 2 Division of Veterinary Clinical Sciences, University of Edinburgh, Easter Bush Veterinary Centre, Roslin, EH25 9RG, UK; 3 Current address: DSTL, Porton Down, Salisbury, Wiltshire, SP4 0JQ, UK

## Abstract

**Background:**

*Trichuris muris *in the mouse is an invaluable model for infection of man with the gastrointestinal nematode *Trichuris trichiura*. Three *T. muris *isolates have been studied, the Edinburgh (E), the Japan (J) and the Sobreda (S) isolates. The S isolate survives to chronicity within the C57BL/6 host whereas E and J are expelled prior to reaching fecundity. How the S isolate survives so successfully in its host is unclear.

**Results:**

Microarray analysis was used as a tool to identify genes whose expression could determine the differences in expulsion kinetics between the E and S *T. muris *isolates. Clear differences in gene expression profiles were evident as early as day 7 post-infection (p.i.). 43 probe sets associated with immune and defence responses were up-regulated in gut tissue from an E isolate-infected C57BL/6 mouse compared to tissue from an S isolate infection, including the message for the anti-microbial protein, angiogenin 4 (Ang4). This led to the identification of distinct differences in the goblet cell phenotype post-infection with the two isolates.

**Conclusion:**

Differences in gene expression levels identified between the S and E-infected mice early during infection have furthered our knowledge of how the S isolate persists for longer than the E isolate in the C57BL/6 mouse. Potential new targets for manipulation in order to aid expulsion have been identified. Further we provide evidence for a potential new marker involving the acidity of the mucins within the goblet cell which may predict outcome of infection within days of parasite exposure.

## Background

Studies of *Trichuris muris *focus on one particular isolate, the Edinburgh (E) isolate. Infections of resistant mice, such as BALB/c, with the E isolate results in a protective Th2 response. Susceptibility to infection is associated with the host mounting a IFN-γ-dominated response inappropriate for worm expulsion and this is seen in mouse strains such as AKR [1-3].

However, other laboratory isolates of *T. muris *exist, the Japan (J) isolate, sub-cultured from the E isolate and the Sobreda (S) isolate discovered in Portugal. Interestingly, these isolates provoke different immune responses within the same host [4,5], such as the C57BL/6 mouse strain. In the C57BL/6 mouse the S isolate is able to survive to chronicity, whereas the other two isolate are expelled prior day 21 p.i [6,7]. This therefore, gives us a rare opportunity to study innate and adaptive immune responses to *T. muris *in the context of a resistant or susceptible outcome within one mouse strain without altering worm burden levels. The only other such model available involves manipulating egg dose to generate high or low dose infections and thus resistance (Th2) or susceptibility (Th1) [8]. It has been previously reported that the S isolate survival, within the C57BL/6 mouse, is associated with a dampened effector Th2 response and an increased Th1 responses [6,7].

Little is known however, about the underlying mechanisms evolved by the S isolate to enhance its survival within the host. Data from our laboratory suggests that the S isolate has evolved methods of manipulating the hosts T regulatory cell arm of the immune response[9], and the responses of key antigen presenting cells to parasite antigens[10]. However differences in gene expression locally in gut tissue p.i. have not been analysed, despite the fact that they may underlie subsequent infection outcome.

Microarray analysis is a useful tool to look at global gene expression changes and indeed has been utilised to usefully inform research in many infections and diseases including *Helicobacter pylori *infection [11,12] and inflammatory bowel disease [13]. Interestingly, data already published from our laboratory has used oligonucleotide microarrays to determine gene expression changes in either resistant (BALB/c) or susceptible (AKR) mice infected with the E isolate of *T. muris *at 19 or 60 days post infection (p.i). Results indicated that AKR mice had a Th1- dominated mucosa, with up-regulated expression of genes associated with IFN-γ and BALB/c mice up-regulated the expression of genes coding for potential anti-parasitic proteins including intelectin and angiogenins [14]. The association of IFN-γ with susceptibility during a *T. muris *infection has also been shown via reverse transcription (RT)-PCR analyses [15] and its functional importance revealed by blocking studies [16].

Here we analyse gut tissue, from C57BL/6 mice infected with either the E isolate or the S isolate. A time point of 7 days p.i was chosen, as it is a time point where the host will not have expelled either of the isolates (D'Elia *et al*. unpublished data), yet early gene expression changes may be occurring which later determine whether the parasite is expelled or not. Data presented here highlights the possible importance of two genes in particular, indoleamine 2,3-dioxygenase (*INDO*) and angiogenin 4 (Ang4). *INDO *is a gene whose expression is up-regulated by both isolates and may aid early survival within the host. Ang4 is a gene whose expression is differentially regulated by the two isolates and may determine outcome of infection within the C57BL/6 host. Further, histological analysis of goblet cells demonstrated differences in their mucin content p.i with the E or S isolates of *T. muris*. Changes in Ang4 expression and/or other genes described here may explain the alterations of mucin acidity within the goblet cell and these changes may contribute to S isolate survival within the C57BL/6 host.

## Results

### Enhanced survival of the S isolate of *T. muris *compared to the E isolate in C57BL/6 mice

**Figure 1:**
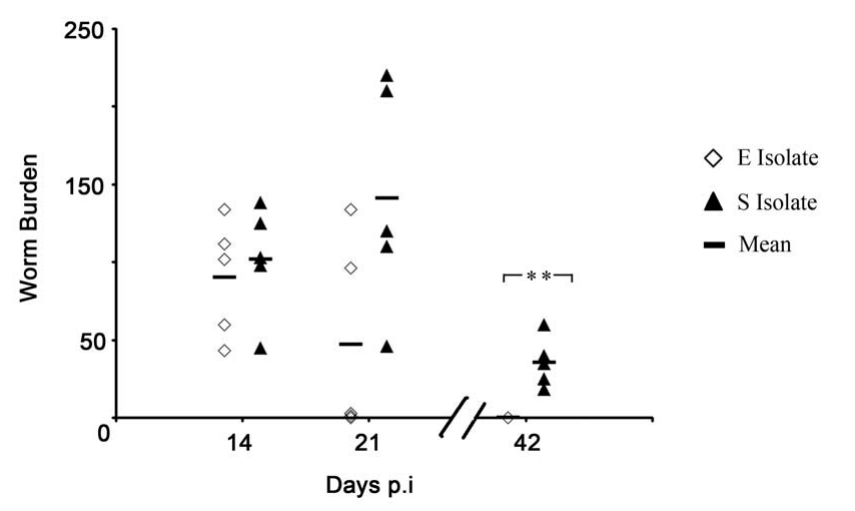
**E and S isolate infection in C57BL/6 mice**. C57BL/6 mice were infected with ~200 embryonated eggs from either the E or the S isolate of *T. muris*. At days 14, 21 and 42 p.i. the mice were sacrificed, caecae and proximal colons were removed and the number of worms counted. Each symbol represents an individual animal (5 mice per group) and the line represents the mean. **, significant difference between the E isolate and S isolate-infected mice (Mann Whitney U test, p < 0.01).

As previously reported [7], the S isolate of *T. muris *is able to persist for longer and indeed reach fecundity in a C57BL/6 mouse unlike the E isolate of *T. muris*. As shown in Fig. 1, worm expulsion is clearly evident at d21 p.i in an E isolate infection with only two mice harbouring high number of worms. In contrast, all five mice infected with the S isolate have high numbers of worms at d21 p.i and still have worms present at d42 p.i where as, at this time point, all five C57BL/6 mice have expelled the E isolate (p = 0.0079, Mann-Whitney test).

### Commonality and differences in RNA expression profiles following infection with the E or S isolates of *T. muris*

**Figure 2:**
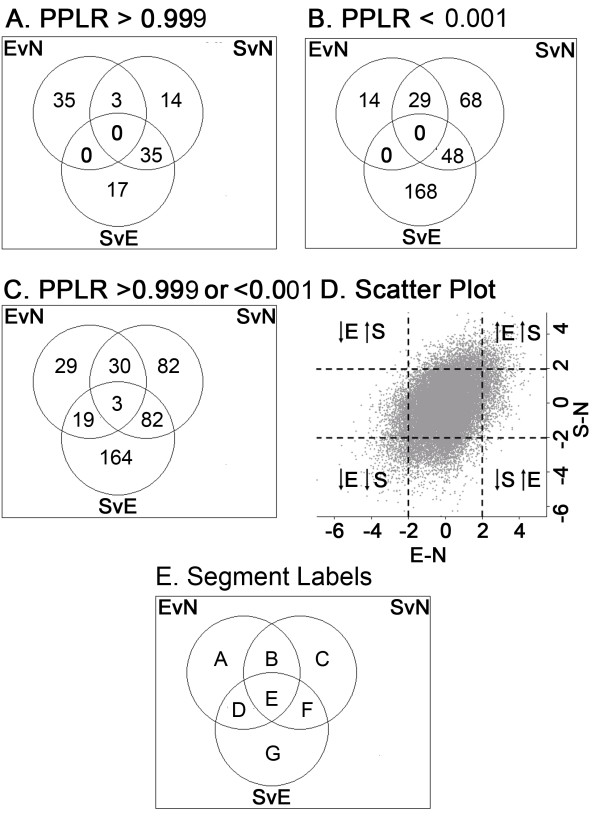
**Analysis of microarray data of Trichuris infected gut tissue with either the E isolate or S isolate shown in Venn Diagrams and scatter plots**. PPLR values were calculated for E isolated-infected gut tissue compared to naïve (uninfected) gut tissue (EvN), S isolated-infected gut tissue compared to naïve gut tissue (SvN) and S isolated-infected gut tissue compared to E isolate-infected gut tissue (SvE). Distribution of probe sets with a PPLR value greater than 0.999 (A), PPLR less than 0.001 (B) and PPLR greater than 0.999 and/or less than 0.001 (C) in any one of the three comparisons (EvN, SvN or SvE) are shown in Venn diagrams. The scatter plot (D) shows the average log_2_-fold changes in probe set expression in gut tissue from E isolate-infected C57BL/6 mice at day 7 compared to that from uninfected C57BL/6 mice (*x *axis) versus the average log_2_-fold changes in probe set expression in gut tissue from S isolate C57BL/6 mice at day 7 compared to that from uninfected C57BL/6 mice (*y *axis). Gut tissue was pooled from five individual animals within a group, and three independent infection experiments were run. The average log_2_-fold changes presented represent values calculated from all three infection experiments.

Mice infected with either the E or S isolates of *T. muris *were sacrificed at d7 p.i. Microarray analysis was carried out at this time point, prior to expulsion of either isolates to identify changes in gene expression which might reflect the early immune interactions which underlie the subsequent differences in survival. Microarray data from RNA extracted from three gut samples (naïve C57BL/6 mice, E isolate-infected C57BL/6 mice d7 p.i and S isolate-infected C57BL/6 mice d7 p.i), done in triplicate, was analysed by the PUMA Bayesian statistical method [17]. The probability of positive log-ratio (PPLR) statistical scores were generated for each probe set by two-way comparisons between the three sample groups. PPLR values range from 0 to 1, with values closest to 0 representing the most significantly down-regulated probe sets and values closest to 1, representing the most significantly up-regulated probe sets. Values of 0.5 represent no significant change [17,18]. Venn diagrams were generated to show the distribution of the probe sets. Probe sets with a probability of positive log-ratio (PPLR) value greater than 0.999 (significantly up-regulated) in at least one of the comparisons (probe set expression of gut tissue from an E isolate infection versus that of naïve gut tissue [EvN], probe set expression of gut tissue from an S isolate infection versus that of naïve gut tissue [SvN] and probe set expression of gut tissue from an S isolate infection versus that of gut tissue from an E isolate infection [SvE]) indicate that 35 probe sets are uniquely up-regulated when comparing the gene expression profile of gut tissue from a naïve mouse to the gene expression profile of gut tissue from an E isolate-infected mouse (EvN). When comparing SvN, 14 probe sets are unique, interestingly however, 3 probe sets are up-regulated in both an E isolate infection and an S isolate infection. Importantly there were 17 probe sets that were uniquely up-regulated when comparing the gene expression profiles of gut tissue taken from mice infected with the two isolates - SvE (Fig. 2A). The number of probe sets associated with a significantly down-regulated PPLR value (0.001) was higher than the number for up-regulated probe sets. 168 probe sets alone were significantly down-regulated in an S isolate infection compared to an E isolate infection- SvE (Fig. 2B).

Combining probe sets that were either significantly up-regulated (0.999) or significantly down-regulated (0.001) in at least one of the comparisons (EvN, SvN and SvE) generated a list of 409 probe sets. 164 of these probe sets were unique to SvE (Fig. 2C and Table 1G). A total of 29 genes were significantly up-regulated or down-regulated in gut tissue p.i with the more commonly studied E isolate compared to an uninfected (Naïve) mouse (Fig. 2C and Table 1A). Only 3 genes, however, were common in all three comparisons (EvN, SvN and SvE) (Fig. 2C and Table 1E). Examples of the 409 probe sets are shown in Table 1, with the table split into sections representing the segments shown in Fig. 2E (full list of probe sets available in additional data file 1).

**Table 1:** Selection of probe sets from each section of the Venn diagram with a PPLR greater than 0.999 and/or less than 0.001.

			**PPLR Value**		
			
**Probe Set ID**	**Gene Symbol**	**Gene Description**	**E v N**	**S v N**	**S v E**
***A. Probe sets of PPLR > 0.999 or < 0.001 in E v N only***					

1417655_a_at	Ars2	arsenate_resistance_protein_2	0.999501	0.887027	0.005792

1418652_at	Cxcl9	chemokine_(C-X-C_motif)_ligand_9	0.999702	0.997352	0.083513

1449102_at	Ebf2	early_B-cell_factor_2	9.14E-04	0.001533	0.670498

1435906_x_at	Gbp2	guanylate_nucleotide_binding_protein_2	0.999957	0.920883	0.003407

1419060_at	Gzmb	granzyme_B	0.999274	0.834789	0.004865

1425477_x_at	H2-Ab1	histocompatibility_2,_class_II_antigen_A,_beta_1	0.999653	0.917835	0.009449

1420437_at	Indo	indoleamine-pyrrole_2,3_dioxygenase	0.999996	0.998805	0.057528

1447927_at	Mpa2l	macrophage_activation_2_like	0.999643	0.721523	0.002485

***B. Probe sets of PPLR > 0.999 or < 0.001 in E v N and S v N***					

1425519_a_at	Cd74	CD74_antigen_(invariant_polypeptide_of_major_histo compatibility_complex,_class_II_antigen-associated)	1	0.999649	0.002733

1456612_at	Il17d	Interleukin_17D	4.12E-06	4.19E-06	0.535351

1427615_at	Itga4	integrin_alpha_4	8.56E-04	5.59E-04	0.422541

1428056_at	Klra7	Killer_cell_lectin-like_receptor,_subfamily_A,_member_7	5.32E-04	6.98E-04	0.561618

1430132_at	Krt28	keratin_28	9.00E-04	2.22E-04	0.244397

1441873_at	Prlpe	prolactin-like_protein_E	3.25E-05	3.65E-05	0.605783

1428111_at	Slc38a4	solute_carrier_family_38,_member_4	1.51E-04	3.85E-04	0.6009

1429901_at	Tcba1	T-cell_lymphoma_breakpoint_associated_target_1	2.14E-04	1.20E-04	0.438909

***C. Probe sets of PPLR > 0.999 or < 0.001 in S v N only***					

1419549_at	Arg1	arginase_1,_liver	0.23588	5.87E-04	0.003669

1425532_a_at	Bin1	bridging_integrator_1	0.985409	0.999463	0.941873

1449637_at	Cdh4	cadherin_4	0.001517	4.54E-04	0.293429

1422542_at	Gpr34	G_protein-coupled_receptor_34	0.0022	5.97E-04	0.51351

1427850_x_at	Igh-VJ558	immunoglobulin_heavy_chain_(J558_family)	0.819554	0.999188	0.995084

1422250_at	Map3k2	mitogen_activated_protein_kinase_kinase_kinase_2	0.790984	0.999799	0.998933

1417051_at	Pcdh8	protocadherin_8	0.704933	0.999094	0.997809

1416576_at	Socs3	suppressor_of_cytokine_signaling_3	0.927402	0.999427	0.989809

***D. Probe sets of PPLR > 0.999 or < 0.001 in E v N and S v E***					

1440832_at	Ang4	angiogenin,_ribonuclease_A_family,_member_4	0.999347	0.119804	3.84E-06

1419684_at	Ccl8	chemokine_(C-C_motif)_ligand_8	0.999999	0.98578	4.66E-04

1447620_at	Cno	cappuccino	0.999021	0.473507	4.52E-04

1426731_at	Des	desmin	0.999964	0.270284	1.93E-06

1435290_x_at	H2-Aa	histocompatibility_2,_class_II_antigen_A,_alpha	1	0.928626	4.61E-08

1419043_a_at	Iigp1	interferon_inducible_GTPase_1	0.999969	0.644236	2.52E-04

1449490_at	Mbd4	methyl-CpG_binding_domain_protein_4	0.999147	0.449218	1.50E-04

1423505_at	Tagln	transgelin	0.999831	0.031314	1.35E-08

***E. Probe sets of PPLR > 0.999 or < 0.001 in E v N and S v E and S v N***					

1455106_a_at	Ckb	creatine_kinase,_brain	0.999225	6.03E-04	2.32E-10

1418240_at	Gbp2	guanylate_nucleotide_binding_protein_2	1	0.999995	1.49E-07

1449009_at	Tgtp	T-cell_specific_GTPase	1	0.999989	4.83E-11

***F. Probe sets of PPLR > 0.999 or < 0.001 in S v N and S v E***					

1425103_at	Ace2	angiotensin_I_converting_enzyme_(peptidyl- dipeptidase_A)_2	0.275223	0.999142	0.999784

1416330_at	Cd81	CD_81_antigen	0.599285	4.84E-06	1.05E-06

1457644_s_at	Cxcl1	chemokine_(C-X-C_motif)_ligand_1	0.378865	0.999467	0.999866

1416685_s_at	Fbl	fibrillarin	0.873786	1.01E-06	1.11E-09

1416221_at	Fstl1	follistatin-like_1	0.716963	1.49E-04	1.05E-05

1415988_at	Hdlbp	high_density_lipoprotein_(HDL)_binding_protein	0.531698	5.56E-04	4.14E-04

1447692_x_at	Map3k7ip1	mitogen-activated_protein_kinase_kinase_kinase _7_interacting_protein_1	0.321227	0.999609	0.999863

1450641_at	Vim	vimentin	0.727751	5.04E-05	2.09E-06

***G. Probe sets of PPLR > 0.999 or < 0.001 in S v E only***					

1443341_at	Anxa4	Annexin_A4	0.994522	0.054991	3.27E-06

1460218_at	Cd52	CD52_antigen	0.979891	0.167083	9.70E-04

1418457_at	Cxcl14	chemokine_(C-X-C_motif)_ligand_14	0.931634	0.008835	1.23E-04

1422412_x_at	Ear3	eosinophil-associated,_ribonuclease_A_family,_ member_3	0.947348	0.011739	3.24E-04

1423407_a_at	Fbln2	fibulin_2	0.919939	0.025244	2.66E-04

1457346_at	Foxp1	Forkhead_box_P1	0.998173	0.287033	3.68E-04

1427351_s_at	Igh-6	immunoglobulin_heavy_chain_6_(heavy_chain_of_IgM)	0.974353	0.029092	6.25E-05

1454665_at	Irf2bp2_///_LOC672960	interferon_regulatory_factor_2_binding_protein_2_///_ similar_to_interferon_regulatory_factor_2_binding_ protein_2	0.660352	0.003366	8.80E-04

1415961_at	Itm2c	integral_membrane_protein_2C	0.699876	0.003609	4.85E-04

1451407_at	Jam4	junction_adhesion_molecule_4	0.130394	0.98967	0.999784

1449989_at	Mcpt2	mast_cell_protease_2	0.997228	0.27691	3.33E-04

1443037_at	Nptn	Neuroplastin	0.971795	0.087496	5.36E-04

1445827_at	Prkcbp1	Protein_kinase_C_binding_protein_1	0.952881	0.098262	9.82E-04

1456262_at	Rbm5	RNA_binding_motif_protein_5	0.995795	0.026747	1.31E-06

1417654_at	Sdc4	syndecan_4	0.78476	0.003683	2.83E-04

1447034_at	Tnpo1	Transportin_1	0.932695	0.022353	2.24E-04

A = Probe sets of PPLR > 0.999 or < 0.001 in E isolate-infected gut tissue compared to naïve gut tissue only, B = Probe sets of PPLR > 0.999 or < 0.001 in E isolate-infected gut tissue compared to naïve gut tissue and S isolate-infected gut tissue compared to naïve gut tissue, C = of PPLR > 0.999 or < 0.001 in S isolate-infected gut tissue compared to naïve gut tissue only, D = Probe sets of PPLR > 0.999 or < 0.001 in E isolate-infected gut tissue compared to naïve gut tissue and S isolate-infected gut tissue compared to E isolate-infected gut tissue, E = Probe sets of PPLR > 0.999 or < 0.001 in E isolate-infected gut tissue compared to naïve gut tissue and S isolate-infected gut tissue compared to E isolate-infected gut tissue and S isolate-infected gut tissue compared to naïve gut tissue, F = Probe sets of PPLR > 0.999 or < 0.001 in S isolate-infected gut tissue compared to naïve gut tissue and S isolate-infected gut tissue compared to E isolate-infected gut tissue, G = Probe sets of PPLR > 0.999 or < 0.001 in S isolate-infected gut tissue compared to E isolate-infected gut tissue only.

A scatter plot was generated to show the general profile of the microarray data set. Log fold change is shown for S isolate infection over naïve (y axis) vs. log fold change of E isolate infection over naïve (x axis). The majority of probe sets fit within the centre quadrant, representing probe sets with small changes between the isolates. The four quadrants on the extremities of the scatter plot identify the probe sets that may be of interest, i.e. up or down in both the E isolate-infected gut and the S isolate-infected gut compared to naive or up in one isolate infection and down in the other isolate compared to naïve (Fig 2D).

### Microarray probe sets associated with immune response respond similarly in the gut of an S isolate-infected mouse to an un-infected (naïve) mouse

**Figure 3:**
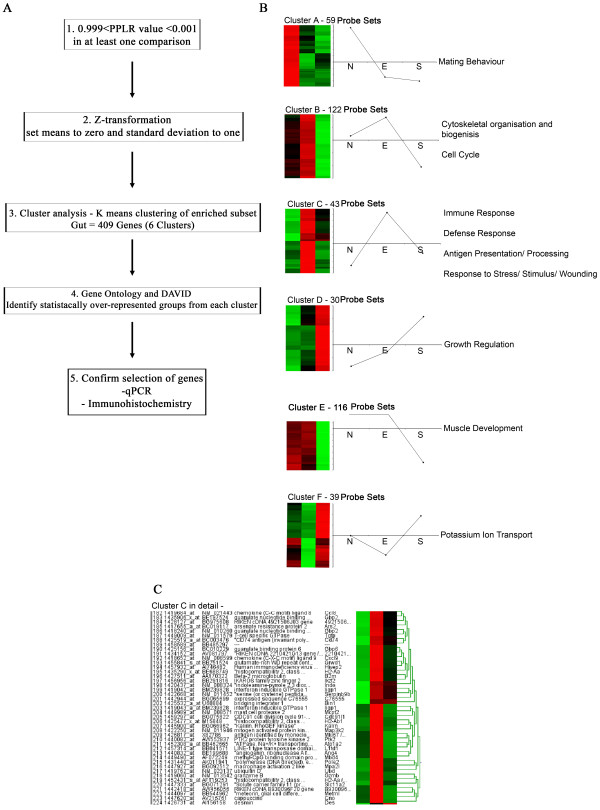
**Clusters, profiles and Gene Ontology groupings of the 409 probe sets that had a PPLR value greater than 0.999 and/or less than 0.001 in at least one of the three comparisons (EvN, SvN or SvE)**. A: Flow chart showing method of generating the clusters from the probe sets. B: The 409 genes were assigned to one of six distinct clusters using k-means clustering algorithms. Data for each cluster are represented with a z-transformed Eisen colour plot with the number of genes in each cluster shown. Red and green indicate positive and negative change from zero, respectively, with colour intensity indicating the degree of deviation. Profiles of the z-transformed data (for each probe set, the mean set to 0 and SD to 1) for each of the experimental groups are shown next to the Eisen plot. The most significantly overrepresented Gene Ontology terms are then shown for the genes within each cluster. C: Expanded version of cluster C indicating the 43 probe sets, showing probe set number, name and symbol along with Eisen plot.

The 409 probe sets that had a PPLR value greater than 0.999 or less than 0.001 in at least one of the comparisons (EvN, SvN and SvE) underwent clustering analysis (Fig. 3A). Six distinct clusters were generated and probe sets from each cluster were subjected to expression analysis systematic explorer (EASE) online tool and DAVID [19,20]. For each cluster, overrepresented gene ontology (GO) groups were identified (Fig 3B).

Cluster A contained 59 probe sets associated with mating behaviour but respond in a similar pattern for both an E isolate infection and an S isolate infection. In cluster B, the S isolate down-regulates 122 probe sets associated with cell cycle and cytoskeletal organisation compared to naïve and E isolate probe sets. In cluster C, containing 43 probe sets, the S isolates expression levels in the gut are similar to naïve levels, whereas the E isolate over express these probe sets. Interestingly, these 43 probe sets are associated with GO groups for immune response, defence response, antigen presentation and response to stress. In cluster D, the S isolate infection causes an increase in gene expression for probe sets associated with growth regulation but down-regulates genes associated with muscle development in cluster E. In both clusters D and E, the E isolate and naïve samples are responding similarly. The final cluster, F, shows no change in naïve levels, a down-regulation with an E isolate infection and an up regulation with the S isolate. These 39 probe sets were associated with potassium ion transport (Fig. 3B). Cluster C is expanded and shown in detail in Fig. 3C. Genes include Angiogenin 4 (Ang4), indoleamine-pyrrole 2,3 dioxygenase (*INDO*), chemokine ligand 8 (CCL8) and interferon inducible GTPase 1 (Iigp1).

### Confirmation of genes from cluster C; angiogenin 4 and indoleamine-pyrrole 2,3 dioxygenase

Given that we had previously demonstrated a correlation between Ang4 expression and resistance and *INDO *expression and susceptibility in a study using different strains of mouse infected with the E isolate [14] we selected these two genes to corroborate our microarray data by qPCR and immunohistochemistry.

**Figure 4:**
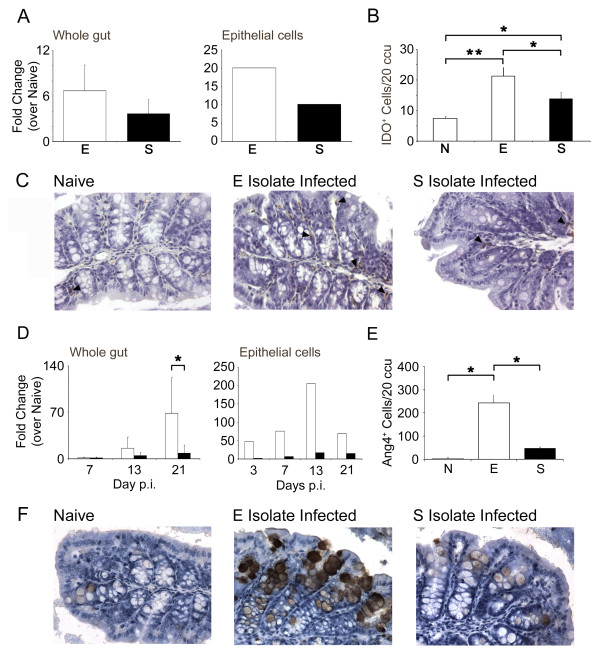
**Quantification of gene expression change by qPCR and Immunohistichemistry for Angiogenin 4 and indoleamine 2,3-dioxygenase**. Gene expression levels for *INDO *in whole gut and epithelial cells (A) comparing E isolate (white bars) to S isolate (black bars) as a fold change over naïve/unstimulated. IDO^+ ^cells were detected in proximal colon tissue sections via immunohistochemistry and data presented as mean number of IDO^+ ^cells per 20 ccu (B). Example of IDO staining (×200 magnification) in naïve, E isolate- and S isolate-infected mice at d7 p.i, with examples of positive cells identified by black arrowheads (C). Gene expression levels for Ang4 in whole gut and epithelial cell fraction (D) comparing E isolate-infected mice (white bars) to S isolate-infected mice (black bars) as a fold change over naïve. *, significant difference between the E isolate and S isolate-infected mice (p < 0.05). Ang4^+ ^cells were detected in proximal colon tissue sections via immunohistochemistry and data presented as mean number of Ang4^+ ^cells per 20 ccu (E). Example of Ang4 staining (×200 magnification) in naïve, E isolate- and S isolate-infected mice at d21 p.i (F). Experiments were performed at least twice and data shown is a representative experiment. Values represent the mean ± SD for 3-4 mice per group.

*INDO *was a highly expressed gene for both E and S isolate compared to naïve (PPLR value = 0.99999, 0.998805 respectively). However when comparing SvE the PPLR value implied that the E isolate transcribed more *INDO *than the S isolate (PPLR = 0.057528). These findings were confirmed by qPCR on RNA extracted from the gut and the epithelial cell fraction (Fig. 4A) (Whole gut - E isolate = 6.68 ± 1.88, S isolate = 3.65 ± 3.4; pooled epithelial cells - E isolate = 20.03, S isolate 10.14).

Although qPCR revealed a non-significant elevation in *INDO *in the gut and epithelial cell fraction of mice infected with the E isolate compared to the S isolate, analysis of IDO at the protein level revealed significantly higher numbers of positive staining cells in an E isolate infection. Thus, infection with either isolate resulted in significantly increased levels of IDO^+ ^cells compared to naïve (E, p < 0.01; S, p < 0.05), with the E isolate infection provoking higher numbers compared to infection with the S isolate (p < 0.05) (Fig. 4B and 4C).

By microarray, gene expression of Ang4 was higher in the gut of mice infected with the E isolate compared to the levels seen in the gut of S isolate-infected C57BL/6 mice (PPLR value = 3.84E-06, Table 1). Ang4 expression was also elevated in whole gut and epithelial cell fractions derived from E-infected mice compared to S-infected mice as identified by qPCR (Fig. 4D). Further, immunohistochemical staining revealed significantly more Ang4^+ ^cells at day 21 p.i. in E isolate-infected mice (Fig. 4E and 4F).

### Increased numbers and acidity of goblet cells in C57BL/6 mice infected with the E isolate compared to the S isolate of *T. muris*

Quantification of Ang4 expression has previously focused on small intestinal Paneth cells as the cellular source [21]. Fig. 4D shows higher expression of Ang4 in the epithelial cell fraction and in Fig. 4F this expression can clearly be seen to localise to the goblet cell suggesting that as no Paneth cells are present in the large intestine, the goblet cell is the most likely source.

**Figure 5:**
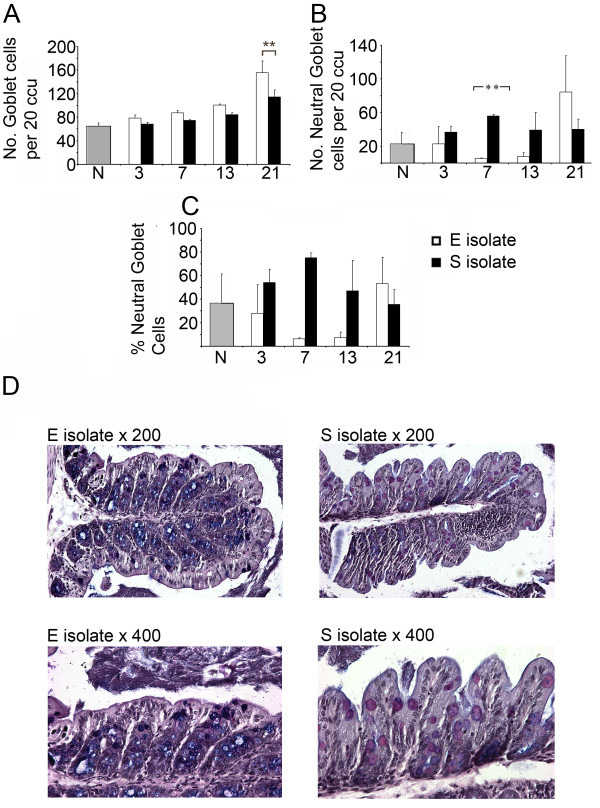
**Changes in number and acidity of goblet cells in E isolate-infected C57BL/6 mice compared to S isolate-infected C57BL/6 mice**. C57BL/6 mice were infected with either the E or S isolates of *Trichuris muris*. At each time point mice infected with either the E or S isolate were sacrificed and goblet cells were detected in proximal colon tissue sections via immunohistochemistry. Data are presented as mean number of goblet cells per 20 ccu (A), mean number of neutral (stain pink following alcian blue and periodic acid treatment) goblet cells per 20 ccu (B) and percentage of neutral goblet cells (C). Examples of goblet cell staining (×200 and ×400 magnification) in E isolate-infected or S isolate-infected mice at d7 p.i (D). *; p < 0.05 and **; p < 0.01 for comparison between the E isolate and S isolate-infected mice (Mann Whitney U test). Experiments were performed at least twice and data shown is a representative experiment. Values represent the mean ± SD for five mice per group.

Therefore, goblet cell staining was carried out on proximal colon sections from naïve, E isolate-infected and S isolate-infected C57BL/6 mice. Total numbers of goblet cells per 20 colonic crypt units (ccu) is shown throughout an infection time course (Fig. 5A). At day 21 p.i an E isolate-infected mice had significantly higher number of goblet cells compared to mice infected with the S isolate (p < 0.05 Mann Whitney U test). Following infection with either the E or S isolate total numbers of goblet cells increased steadily over the full time course from naïve levels.

An interesting phenotypical change in the acidity of mucins was within the goblet cells was evident between the infections with the different isolates. At d7 p.i mice infected with the S isolate of *T. muris *had significantly higher numbers of goblet cells containing neutral (pink) mucins compared to mice infected with the E isolate (Fig. 5B) (p < 0.01 Mann Whitney U test). When these values are converted to percentages of total goblet cell numbers, the pattern is exaggerated at d7 and d13 p.i (Fig. 5C). Examples of typical sections from mice harbouring either the E isolate or S isolate, stained for goblet cells, taken on d7 p.i are shown in Fig. 5D at two magnifications (×200 and ×400) clearly highlight the acidic mucin differences in the goblet cells.

Acidity and/or neutrality of goblet cells are determined by the addition or removal of sugars on mucins by transferases. Increased transferase function would cause increased acidity of mucins within the goblet cells. Table 2 indicates possible transferases that could be causing the phenotypical changes of the goblet cell mucins seen between the two isolates at d7 p.i from the whole gut microarray analyses. Thus, analyses of gene expression levels in whole gut tissue from S and E infected mice reveals a down regulation of all four transferases in tissue from mice infected with the S isolate compared to the E isolate.

**Table 2:** Selections of probe sets which may explain differences associated with neutral goblet cells seen in S isolate-infected mice.

			**PPLR**			**Fold Change**
			
**Probe Set ID**	**Gene Symbol**	**Gene Description**	**E v N**	**S v N**	**S v E**	**S v E**
1420903_at	St6galnac3	ST6 (alpha-N-acetyl-neuraminyl-2,3-beta-galactosyl-1,3)-N-acetylgalactosaminide alpha-2,6-sialyltransferase 3	0.7554	0.1668	0.0473	-3.7792

1425668_a_at	St3gal4	ST3 beta-galactoside alpha-2,3-sialyltransferase 4	0.8429	0.4391	0.1049	-1.4277

1442929_at	St8sia5	ST8 alpha-N-acetyl-neuraminide alpha-2,8-sialyltransferase 5	0.7053	0.2824	0.1351	-3.0470

1443740_at	St3gal6	ST3 beta-galactoside alpha-2,3-sialyltransferase 6	0.1933	0.0122	0.0629	-6.5657

PPLR values for all three comparisons (EvN, SvN or SvE) and fold change in gene expression levels of probe sets from the gut of an S isolate-infected mouse compared to expression levels in an E isolate-infected gut are shown along with probe set identification (ID) number, gene symbol and gene description.

## Discussion

The intimate interactions between hosts and parasites have been studied for many years [22]. The ability of helminths, in particular *T. muris *to affect the immune environment within the host has a broad associated literature [23-25]. Genome wide approaches to understand the changes occurring in the host at a gene expression level have been utilised in many infectious models and diseases [26,27]. Indeed microarray and RT-PCR analysis have been used to explore the responses of mouse strains infected with *T. muris *[14,15]. However, these experiments use different mouse strains in order to compare the gene expression profiles associated with resistance or susceptibility. Here we use one mouse strain, C57BL/6, and different *T. muris *isolates to achieve susceptibility or resistant outcomes. In addition we look early in infection to identify changes in gene expression, that could later determine susceptibility or resistance of the host.

We demonstrate that the S isolate of *T. muris*, which is able to persist chronically in a C57BL/6 host, provokes a different gene expression profile in gut tissue compared to a C57BL/6 mouse infected with the E isolate, which is ultimately expelled. These changes are already apparent by d7 p.i. prior to E isolate expulsion. In particular, differences in 43 probe sets associated with GO groups - immune response, defence response, antigen presentation and response to stress were identified.

Interestingly the expression of genes from an S isolate infected gut resemble those seen in a naïve (uninfected) gut, whereas there was an elevation in the expression of the genes represented by the 43 probe sets during an E isolate infection. This data would imply that even at d7 p.i., the S isolate is dampening down expression of genes that would normally be associated with expulsion of the parasite. Microarray analyses on gut tissue from different strains of mice infected with the E isolate had previously identified differences in the expression of *INDO *and Ang4. As these two genes were also identified as being differentially expressed in this current study we investigated their expression further.

Indoleamine 2,3-dioxygenase plays an important part in the kynurenine pathway where it metabolises tryptophan which has been associated with controlling parasite growth in a number of infections [28-30]. IDO also has immunoregulatory roles [14,31,32], indeed its ability to dampen cell proliferation [31] may extend to the control of gut epithelial cell turnover and thus delayed or absent worm expulsion given the importance of epithelial cell turnover in the expulsion of *T. muris *[33]. As the expression of IDO was elevated in gut tissue from both E and S isolate-infected mice its presence may reflect a less efficient ability, rather than a complete inability, to expel the parasite.

In contrast, Ang4 expression did differentiate between worm survival and expulsion with expression levels only elevated in E-infected mice. The angiogenins are a family of closely related proteins described as Paneth cell-derived and encoded for, in the mouse, by a gene cluster on chromosome 1 [34]. They belong to the RNase superfamily [35] which includes eosinophil secretory granules proteins which are toxic to some gastrointestinal nematodes [36]. Originally implicated in the growth of tumours, the normal physiological role of the angiogenins was unclear until the demonstration of a role for Paneth cell derived Ang4 as an endogenous anti-microbial protein central in epithelial host defence against gut-dwelling bacteria [21]. Immunohistochemical staining of gut tissue in this study localised Ang4 production to the goblet cell, identifying the cell type as a novel cellular source in the large intestine. This interesting discovery expands on the already extensive literature on the importance of goblet cells in the expulsion of nematode infection, where increased numbers of goblet cells correlate with resistance [37,38].

A goblet cell hyperplasia is reported in the context of many nematode infections including *T. muris*, *T. spiralis*, *H. polygyrus *and *N. brasiliensis *and this is thought to be under the control of a Th2 response [37-39]. Several goblet cell factors have been identified to play an important role in nematode infection including Relm-β and Muc2. Relm-β expression is linked with the production of Th2 cytokines and worm expulsion in *T. muris*, *T. spiralis *and *N. brasiliensis *[40]. Thus effective worm expulsion may be achieved through combined effects of antimicrobial proteins including the angiogenins, Relm-β and indeed the intelectins [14,41].

Goblet cell numbers did not differ quantitatively between E and S isolate infection until d21 p.i. However, the goblet cells were qualitatively strikingly different at d7 p.i. Thus the number of neutral goblet cells present in the gut of an S isolate infected C57BL/6 mouse are significantly higher than that seen in an E isolate infected mouse. By d21 the number of neutral goblet cells is similar for both isolates, if not higher in the E isolate infection. At this time point the majority of E isolate worms have been expelled.

The acidity of goblet cells is determined by the addition and removal of sugars to the mucins within the goblet cell [42]. Transferases are required to do this and Table 2 highlights candidate transferases that may explain the differences seen in the quality of the goblet cells. Increased gene expression of the transferases is seen in the microarray data in the gut of an E isolate-infected mouse compared to an S isolate-infected gut. The increases in gene expression of the transferases listed could explain the increased acidity of the goblet cell mucins seen in an E isolate infection and may even underlie subsequent expulsion.

## Conclusion

The C57BL/6 mouse strain differs in its ability to expel the E and S isolates of *T. muris*. Microarray analysis of gut tissue taken from infected mice just one week p.i. revealed significant differences in gene expression profiles. Thus, under similar levels of parasite exposure in a single mouse strain changes which ultimately correlate with resistance or susceptibility are apparent.

We report an association between the expression of the anti-microbial protein Ang4 and resistance to infection and further identify the goblet cell as a novel cellular source. Qualitative differences in goblet cell mucins early p.i. are also reported and may act as an early predictor of future resistance or susceptibility.

## Methods

### Mice

C57BL6 male mice were obtained from Harlan (Bicester, UK) at 6-8 weeks of age. Mice were specific pathogen free and maintained in sterile conditions in individually ventilated cages by the Biological Services Faculty (BSF), University of Manchester, UK. All work was performed under the regulations of the Home Office Scientific Procedures Act (1986).

### Parasites

Both the E and S isolate of *T. muris *were maintained as previously described [43]. Excretory/secretory (E/S) antigen was collected by incubating adult worms in RPMI media for 4 hrs. Mice were infected orally with around 150-200 embryonated eggs. Worm burden analysis was carried out as previously described [44].

### Sample collection/protocol

C57BL/6 mice were infected with ~200 embryonated eggs from the E isolate or the S isolate of *T. muris*. 7 days p.i. with the E or S isolate of *T. muris*, groups of 5 mice were killed and gut samples were taken from each mouse, including naïve (uninfected) mice. Samples were placed in trizol and snap frozen in liquid nitrogen for RNA extraction or placed in Neutral buffer formalin (NBF) for immunohistochemistry.

Two groups of five mice infected with either the E isolate or the S isolate of *T. muris *were allowed to progress to day 14 p.i to determine worm burden. Three independent infection experiments were run.

### Isolation of intestinal epithelial cells

As previously described, [14]. Briefly, the caecum and approximately 5 cm of colon were removed. The tissue was then slit longitudinally and rinsed in calcium- and magnesium-free Hanks balanced salt solution containing 2% foetal calf serum (FCS) (CMF2%), cut into 1-cm pieces, and placed into ice-cold CMF2%. Samples were washed until supernatant was clear. The tissue was then placed into calcium- and magnesium-free Hanks balanced salt solution containing 10% FCS, 1 mM EDTA, 1 mM dithiothreitol, 100 units/ml penicillin, and 100 μg/ml streptomycin (CMF10%) and incubated at 37°C for 20 min. The supernatant was then passed through a 100-μm cell strainer (Becton Dickinson, Oxford, United Kingdom) and centrifuged at 200 × *g *for 10 min. The cells were resuspended in ice-cold RPMI 1640 (Invitrogen, Carlsbad, CA), 5% Foetal calf serum (FCS), 100 U/ml penicillin and 100 μg/ml streptomycin, 1% L-glutamine, 0.1% MTG (Sigma Aldrich, St Louis, MO). The cell concentration determined using a CASY-1 Coulter Counter. The final cell concentration was adjusted to give 5 × 10^6 ^cells/ml. An aliquot was then taken for RNA extraction.

### RNA extraction

Gut Samples were removed from -80°C freezer and allowed to thaw on ice. Each sample was placed into a sterile falcon tube and homogenised using an Ultra -Turrax (T25 Basic) homogeniser. The homogeniser was cleaned with chloroform, ethanol and 3× DEPC water (Promega) between each sample. The samples were then placed in RNAse-free 1.5 ml eppendorfs. Following tissue homogenisation, cell debris was pelleted by spinning at 13000 rpm for 10 minutes at 4°C. The supernatant was then recovered into a new tube. The samples were incubated at room temperature for 5 minutes. 0.2 ml of chloroform (Sigma Aldrich) was added to the supernatant and samples were shook vigorously for 15 seconds. Samples were then centrifuged for 15 minutes at 4°C at 13000 rpm. The aqueous phase was then transferred to a tube containing 0.5 ml isopropyl alcohol (Sigma Aldrich) and incubated at RT for 10 min. Samples were then centrifuged at 13000 rpm for 10 min at 4°C. The resulting supernatant was removed and 1 ml of 75% ethanol was added. Samples were mixed by vortexing and centrifuged at 12000 rpm for 5 min at 4°C. This was repeated once more. The supernatant was removed and the pellet was air dried for 15-20 minutes. Pellets were resuspended in 50 μl of nuclease free water (Promega). Samples were then incubated at 60°C for 10 minutes. RNA was pooled from five individual animals within a group and each group run as an individual microarray chip. Three independent infection experiments were run and therefore a total of 9 microarray chips were run and analysed.

### DNase treatment

A turbo DNase kit (Ambion Turbo DNA-free) was used to remove all pieces of contaminating DNA. To each 50 μl of RNA, 5 μl of Turbo DNase buffer and 1 μl of DNase enzyme was added. Samples were then incubated at 37°C for 30 minutes. Following incubation, 5 μl of turbo DNase inactivation buffer was added and left for 2 minutes. Samples were then spun down for 1 minute at 13000 g. The supernatant was then placed into a clean 200 μl eppendorf and stored at -80°C. The concentration of RNA was determined via Nanodrop spectrometry (Nanodrop technologies). The quality of RNA was checked on a 2% agarose gel in 0.5× TBE buffer (Invitrogen) with 0.01% Ethidium Bromide. Samples were loaded with Orange G loading buffer (4% sucrose in dH_2_0 + 0.01% Orange G).

### Microarray procedure

RNA quality was checked using the RNA 6000 Nano Assay, and analyzed on an Agilent 2100 Bioanalyser (Agilent Technologies). RNA was quantified using a Nanodrop ultra-low-volume spectrophotometer (Nanodrop Technologies).

Hybridization cocktail was hybridised to HG-U133 PLUS2 oligonucleotide arrays (Affymetrix) according to manufacturer's instructions. Arrays were read using Agilent GeneArray scanner 3000 7G using Affymetrix GCOS (V1.4) software.

### Microarray analysis

Technical quality control was performed with dChip (V2005) [45] Normalisation and expression analysis was done using multi-mgmos [46]. Differential expression between the sample groups (E, S, N) was assessed with a Bayesian method which includes probe-level measurement error when assessing statistical significance [18]. Analysis was performed with the PUMA package in R [17,47].

A gene list of differentially expressed genes (409 probe sets) was created by filtering for probe sets with a probability of positive log-ratio (PPLR) value less than 0.001 (or greater than 0.999) in any of the three-way comparisons between E, S and N samples. This data set was segregated into 6 clusters based on similarity of expression profile across the dataset using a k-means clustering algorithm. Clustering was performed on the means of each sample group (log 2) that had been z-transformed (for each probe set the mean set to zero, standard deviation to 1). K-means clustering was done on the basis of similarity of profiles across the dataset using the "Super Grouper" plug-in of maxdView software (available from )). Functional annotation of the genes was performed using DAVID version 2. ) [19].

### MIAME

Microarray data has been submitted in a MIAME compliant standard to the Array Express database (Experiment E-MEXP-1795, )

### Quantitative real-time polymerase chain reaction (qPCR)

1.0 μg of total RNA was reverse transcribed using Bioscript (Bioline, London, U.K.) in a final volume of 40 μl according to the manufacturer's instructions and stored at -20° until used. Quantitative PCR was performed using SensiMix plus SYBR (Bioline) on an OPTICON DNA engine with OPTICON MONITOR software version 2·03 (Real-Time systems; MJ Research, Hemel Hempstead, UK). Amplification of mRNA encoding 18S was performed to control for the starting amount of cDNA. Expression levels of genes of interest are shown as fold change over that seen in naïve animals after normalization to housekeeping gene levels using the ΔΔC^t ^method. Primers sequences were AGTCCCTGCCTTTGTACACA and GATCCGAGGGCCTCACTAAC for *18S*, CTGCACGACATAGCTACCAGTCTG and ACATTTGAGGGCTCTTCCGACTTG for *IDO *and CTCTGGCTCAGAATGTAAGGTACGA and GAAATCTTTAAAGGCTCGGTACCC for *Ang4*. All sequences are 5'-3' with the sense primer given first.

### Histology

Histological sections were prepared from proximal colon tissue fixed in 10% buffered formalin and embedded in paraffin. 6-μm sections were cut using a microtome and added to gelatin-coated microscope slides. Sections were dewaxed with citroclear and taken to water through decreasing concentrations of ethanol.

For IDO and Ang4 staining slides were washed in PBS and endogenous peroxidase activity was quenched using 0.064 mg/ml sodium azide, 1.5 U/ml glucose oxidase, and 1.8 mg/ml D-glucose (Sigma-Aldrich) in PBS for 20 min at 37°C. Endogenous avidin and biotin binding sites were blocked using a commercial kit according to the manufacturer's instructions (Vector Laboratories). For IDO staining this was followed by a mouse on mouse kit, M.O.M (Vector Laboratories). Briefly, slides were incubated for 1 hr in mouse Ig blocking reagent, 5 minutes in working solution and 30 minutes in primary antibody (MAB5412, MS × Indoleamine 2,3-dioxygenase). For Ang4 staining slides were incubated consecutively with 1.5% donkey serum (Jackson Immunoresearch), sheep anti-mouse Ang4 and biotinylated F(ab')_2 _donkey anti-sheep IgG (Jackson Immunoresearch). Colour development in both cases was by incubation with ABC (Avidin Biotin Complex), DAB (3,3'-diaminobenzidine) and haematoxylin (all from Vector Laboratories).

For goblet cell staining, mucins in goblet cells were stained with 1% alcian blue (Sigma) in 3% acetic acid, washed and treated with 1% periodic acid (Sigma) followed by counterstaining in Mayer's haematoxylin (Sigma). Slides were dehydrated and mounted in aquamount (BDH Laboratory Supplies, Poole, UK). For enumeration of immunohistochemistry and goblet cell staining, the average number of cells from 20 crypts was taken from three different sections per mouse.

### Statistics

Differences between groups were tester by non-parametric methods, the Mann-Whitney U test (two factor comparisons) and Kruskal Wallis test (for more than two factors) with Dunns post test for multiple parameter comparisons. All tests were performed using GraphPad Prism software (San Diego, CA)

## List of abbreviations

Ang4: Angiogenin 4; d: day; EASE: expression analysis systematic explorer; E/S: Excretory/Secretory; GO: gene ontology; *INDO*: Indoleamine 2,3-dioxygenase gene; IDO: Indoleamine 2,3-dioxygenase protein; MIAME: minimum information about a microarray experiment; p.i.: post infection; PPLR: probability of positive log-ratio; qPCR: quantitative real-time PCR.

## Authors' contributions

RD was involved in study design, performed experiments and data analysis, and was involved in manuscript preparation. MLD performed experiments and data analysis and was involved manuscript preparation. LZ was involved in microarray analysis and manuscript preparation. SHW and AP produced the sheep anti-mouse Ang4 antibody. KJE conceived of the study and participated in its design and coordination and helped to draft the manuscript. Both RD and KJE have had full access to all of the data in the study and take responsibility for the integrity of the data and the accuracy of the data analysis.

## Supplementary Material

Additional file 1 **Significantly altered gene probe set expression in the gut following infection with the E or S isolate of *T. muris***. The fold-change values for all 409 probe sets identified with a PPLR value greater than 0.999 or less than 0.001 in at least one of the comparisons (EvN, SvN and SvE) are presented. Click here for file
